# Supplementary material for: Dynamics of organizational culture: Individual beliefs vs. social conformity
Source: PLoS One. 2017 Jun 30;12(6):e0180193. doi: 10.1371/journal.pone.0180193 (PMC5493361; doi:10.1371/journal.pone.0180193)
Supplement: S1 File — Document contains additional clarifications and results. Table A. Central themes of survey. Spectrum of the six central themes on which the survey builds on. Figure A. Power rank histogram of individuals. Histogram of individuals’ power rank, which corresponds to their role and experience within the organization. Figure B. Additional results for entre range of γ. Additional results, with respect with respect to average cognitive coherence and network coherence, for the entire range of γ, γ ∈ [0,1]. (DOCX) [file pone.0180193.s001.docx]

**Supporting Information for Manuscript: Dynamics of Organizational Culture: Individual Beliefs vs. Social Conformity**

**1. Survey Results**

Each participant was presented with a total of thirty question revolving around six central themes, five questions per theme. The participants were then asked to mark their response on a numerical scale, ranging from -100 to +100 for both current and desirable state. Depending on the theme of the question, the two extremes of the scale correspond to different qualitative organisational features – see Table A.

**Table A**: Spectrum of the six central themes on which the survey builds on.

| **Theme** | **Response Spectrum** | | | |
| --- | --- | --- | --- | --- |
|  | Minimum Score | Corresponds to | Maximum Score | Corresponds to |
| 1 | -100 | Process Oriented | +100 | Goal Oriented |
| 2 |  | Relationship Oriented |  | Task Oriented |
| 3 |  | Organization Oriented |  | Professions Oriented |
| 4 |  | Open Oriented |  | Closed Oriented |
| 5 |  | Loose Control |  | Tight Control |
| 6 |  | Policy Governance |  | Customer Governance |

In addition, the survey recorded the rank of each participant by considering a number of relevant individual traits. In particular, two demographics are used to construct the power rank of each individual: (a) whether they are managers, and (b), years of experience. Aspect (b) is further broken down into four sub-categories – (i) less than two years; (ii) between 2 and 5 years; (iii) between 5 and 10 years, and (iv) over 10 years. Each sub-category incrementally increases the rank of an individual, using a linear scale i.e. (b, i) is worth 1 point; (b, ii) is worth 2 points, (b, iii) is worth 3 points etc. The overall power rank is obtained by summing attribute (a) and (b) for each individual – see Fig A.

**
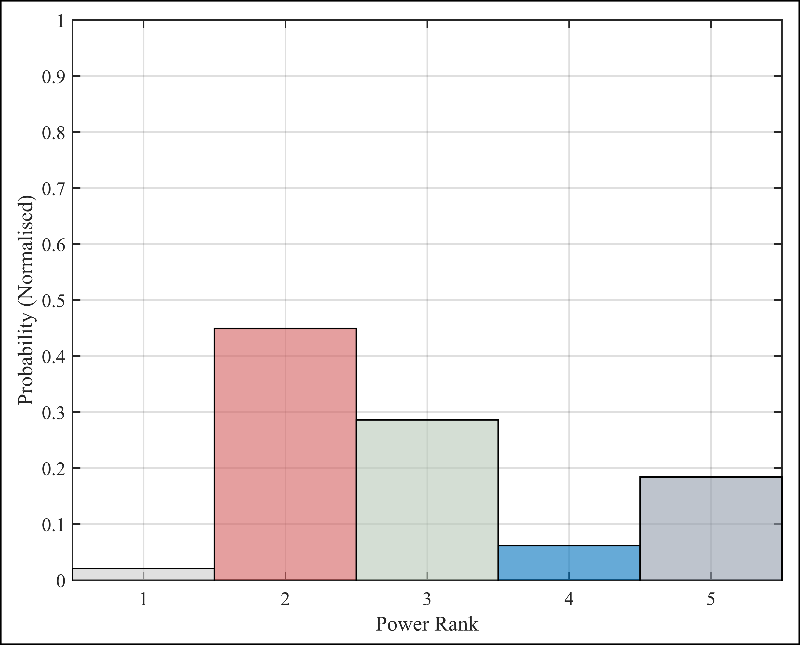
**

**Figure A:** Histogram of individuals’ power rank, which corresponds to their role and experience within the organization.

**2. Initial Model Parameters**

A MATLAB implementation of the algorithm described in ([Clauset, 2005](#_ENREF_1)) is used to construct a random network with a specified number of modules (available for download from <http://strategic.mit.edu/docs/matlab_networks/random_modular_graph.m>.). The specific parameters used are: n=30, c=6; p=0.6; r =0.7, corresponding to number of beliefs; number of modules; overall probability of attachment and proportion of links kept within modules.

**3. Additional Results**

In the spirit of Fig 4, and for the sake of completeness, Fig B presents additional results across the entire range of$\gamma$,$\gamma\in[0,1]$. The core difference between the three main cases previously discussed becomes evident by examining the gradual shifts in both average cognitive coherence and network coherence as γ varies in size. In particular, as the role of peer-pressure increases (i.e. γ increases), network coherence deteriorates faster and reaches lower values, with the lowest value being 0.6. At the same time, average cognitive coherence reverses its initially decreasing trend, to exhibit an increasing trend. This time required for this switch to manifest depends on the value of γ, with higher values leading to a faster switch, in terms of time steps required.

**
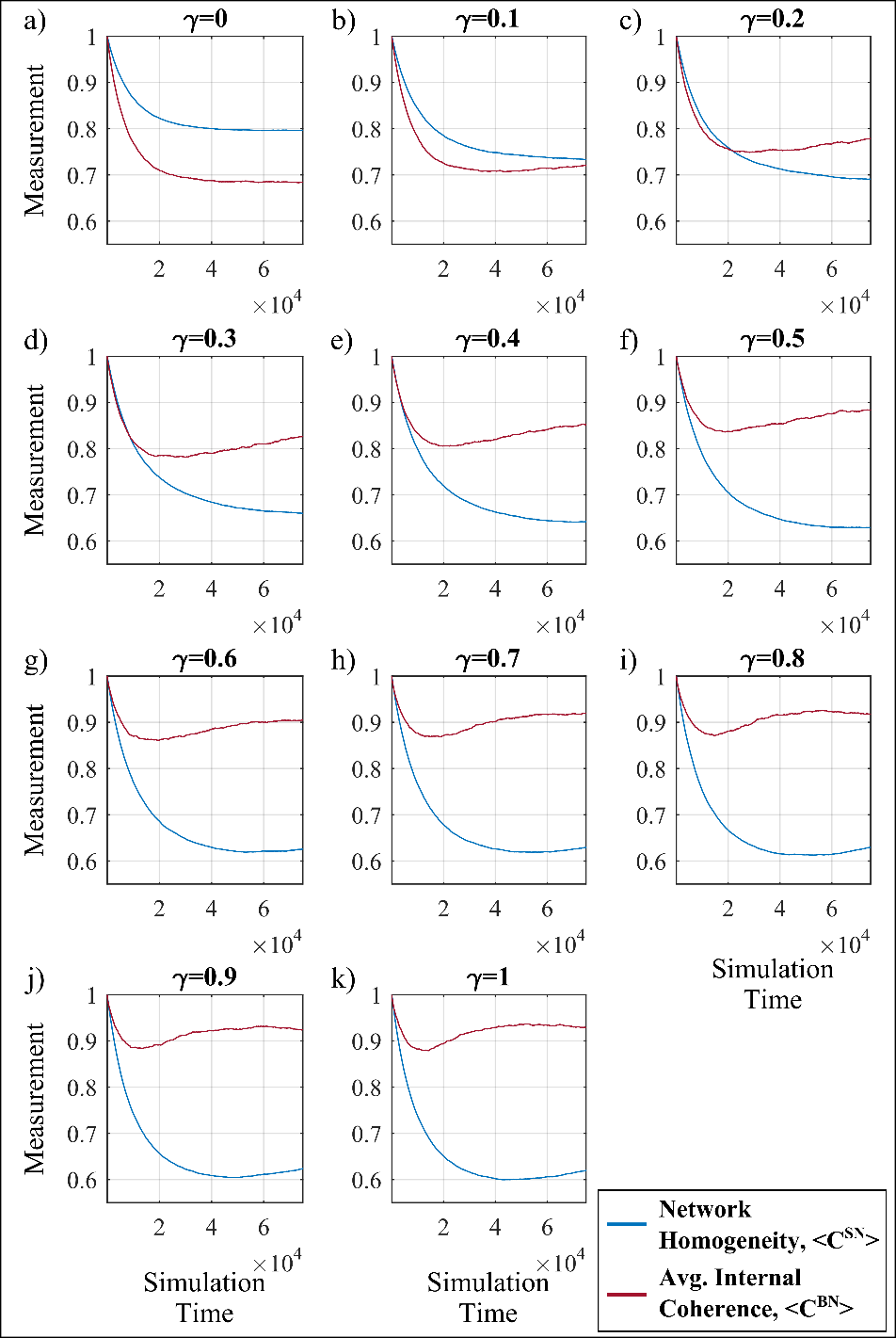
**

**Figure B:** Additional results, with respect with respect to average cognitive coherence and network coherence, for the entire range of$\gamma$,$\gamma\in[0,1]$.

**References**

A. Clauset. 2005. Finding local community structure in networks. *Physical review E*. **72**(2) 026132.
